# Cognitive function after cardiac arrest and temperature management; rationale and description of a sub-study in the Target Temperature Management trial

**DOI:** 10.1186/1471-2261-13-85

**Published:** 2013-10-12

**Authors:** Gisela Lilja, Niklas Nielsen, Hans Friberg, Janneke Horn, Jesper Kjaergaard, Tommaso Pellis, Malin Rundgren, Jørn Wetterslev, Matt P Wise, Fredrik Nilsson, Tobias Cronberg

**Affiliations:** 1 Department of Clinical Sciences, Lund University, Lund, Sweden; 2 Department of Rehabilitation Medicine, Skåne University Hospital, Lund, Sweden; 3 Department of Anesthesia and Intensive Care, Helsingborg Hospital, Helsingborg, Sweden; 4 Department of Anesthesia and Intensive Care, Skåne University Hospital, Lund, Sweden; 5 Department of Intensive Care, Academic Medical Centre, Amsterdam, The Netherlands; 6 Department of Cardiology, The Heart Centre, Copenhagen University Hospital Rigshospitalet, Copenhagen, Denmark; 7 Intensive Care Unit, Santa Maria degli Angeli, Pordenone, Italy; 8 Copenhagen Trial Unit, Centre of Clinical Intervention Research, Copenhagen University Hospital, Copenhagen, Denmark; 9 Adult Critical Care, University Hospital of Wales, Cardiff, United Kingdom; 10 Research and Development centre, Unit for Medical Statistics and Epidemiology, Skåne University Hospital, Lund, Sweden; 11 Department of Neurology, Skåne University Hospital, Lund, Sweden

**Keywords:** Out-of-hospital cardiac arrest, Hypothermia, induced, Resuscitation, Cardiovascular diseases, Brain injuries, Cognition, Memory, Quality of life, Social participation, Caregivers

## Abstract

**Background:**

Mild to moderate cognitive impairment is common amongst long-term survivors of cardiac arrest. In the Target Temperature Management trial (TTM-trial) comatose survivors were randomized to 33°C or 36°C temperature control for 24 hours after cardiac arrest and the effects on survival and neurological outcome assessed. This protocol describes a sub-study of the TTM-trial investigating cognitive dysfunction and its consequences for patients’ and relatives’ daily life.

**Methods/Design:**

Sub-study sites in five European countries included surviving TTM patients 180 days after cardiac arrest. In addition to the instruments for neurological function used in the main trial, sub-study patients were specifically tested for difficulties with memory (Rivermead Behavioural Memory Test), attention (Symbol Digit Modalities Test) and executive function (Frontal Assessment Battery). Cognitive impairments will be related to the patients’ degree of participation in society (Mayo-Portland Adaptability Inventory-4), health related quality of life (Short Form Questionnaire–36v2©), and the caregivers’ situation (Zarit Burden Interview©). The two intervention groups (33°C and 36°C) will be compared with a group of myocardial infarction controls.

**Discussion:**

This large international sub-study of a randomized controlled trial will focus on mild to moderate cognitive impairment and its consequences for cardiac arrest survivors and their caregivers. By using an additional battery of tests we may be able to detect more subtle differences in cognitive function between the two intervention groups than identified in the main study. The results of the study could be used to develop a relevant screening model for cognitive dysfunction after cardiac arrest.

**Trial registration:**

ClinicalTrials.gov: NCT01946932.

## Background

Out of hospital cardiac arrest (OHCA) is common and a major cause of death. Even when resuscitation is successful, there is still an attributable risk for subsequent death [1]. The brain is particularly susceptible to circulatory arrest and hypoxic-ischemic brain injury accounts for approximately 70% of the in-hospital mortality of cardiac arrest (CA) patients admitted to intensive care [2]. Survival with severe neurological disability is uncommon, presumably due to withdrawal of life-supporting treatments (WLST) in patients in whom it is assumed that prognosis is poor [2]. However, studies investigating cognitive function have demonstrated that a large proportion of the survivors are affected by mild to moderate cognitive dysfunction at 3–6 months after cardiac arrest. There is considerable variability in the prevalence of impairment observed in earlier studies, however many of these were limited by small study samples size and the use of tests with insufficient sensitivity to detect mild to moderate cognitive impairment [3]. Some high quality studies show that about half of the CA survivors are left with long-term cognitive impairment, typically involving memory, executive functions and attention [3].

Cognitive dysfunction is known to affect the daily life of patients and their families [3-7]. Most CA survivors perform their basic activities of daily living (ADL) without any significant problems [4]. However, participation in society exemplified by the ability to maintain roles in social, family, home, financial, work or education domains [8] may be considerably affected with as many as 74% of CA survivors having reduced levels of involvement [4]. Participation in the society is related to quality of life and is highly valued by patients, their relatives and society [9,10].

Induced hypothermia was recommended as a neuroprotective treatment after OHCA [11] following clinical trials showing a beneficial effect on neurological outcome [12,13] and survival [13]. However, a systematic review and trial sequential analyses concluded that the evidence was insufficient and that a larger trial to confirm an effect on survival was needed [14]. Whether or not induced hypothermia has an effect on cognitive function has been inadequately investigated. The two key trials [12,13] defined good outcome as Cerebral Performance Categories (CPC) 1–2 [13] or as discharge to home or a rehabilitation centre [12]. Although these outcome measures may detect differences in mortality and severe brain injury, they are clearly insensitive to differentiate between commoner mild to moderate neurological impairment. The CPC score has been found to overestimate favourable outcome, especially when based on hospital discharge records [15-18]. The outcome measure “discharge to a rehabilitation centre” has obvious shortcomings since it may include a range of neurological disability, depending on the evaluation of rehabilitation potential and resources.

Only one randomized study has made a direct comparison between CA patients treated with or without hypothermia using sensitive cognitive tests [16]. In this sub-study of the HACA trial [13], 67% of patients in the hypothermia group had normal or mildly impaired cognition compared to 44% in the control group. This was not statistically significant, possibly due to the small sample size of only 27 hypothermia treated and 18 control patients.

The Target Temperature Management trial (TTM) is a large randomized, parallel group assessor blinded multi-centre study that compares the effects of two strict temperature regimes, 33°C and 36°C for 24 hours after CA. The primary outcome measure is survival, and the secondary outcomes are neurological function and health related quality of life (HRQoL) [19]. This pragmatic trial offers a unique possibility to study cognitive dysfunction in a large international sample of OHCA. The TTM main follow-up component is limited to simple tests and questionnaires. This manuscript presents the rationale and methods of a sub-study of the TTM trial, designed to evaluate cognitive impairment and their impact on participation in society, as well as the next-of-kin’s HRQoL and perception of burden.

### Aims and hypotheses

Our primary aim is to test the hypothesis that cognitive impairment will be less pronounced amongst patients treated with 33°C compared to those treated with 36°C due to a postulated neuroprotective effect of hypothermia, and that both groups of CA patients will have more cognitive impairment than the matched group of controls without CA.

Other hypotheses of this sub-study are that patients with cognitive impairment will have a lower level of participation in the society and a lower HRQoL than CA patients without cognitive impairment and controls. Finally, we hypothesize that cognitive impairment will decrease the caregivers’/relatives’ HRQoL and increase their perception of burden.

## Methods/Design

### Study design

This is a prospective, randomized and pre-registered sub-study of the TTM-trial approved by the TTM-steering group. The TTM-trial started recruitment in November 2010 and reached the inclusion target at 950 patients in January 2013 [19]. At 180 days (± 2 weeks) after cardiac arrest, all TTM survivors attended a face-to-face follow-up to evaluate their neurological function and HRQoL [19]. During this follow-up meeting, patients and their representatives included in the sub-study performed additional assessments relating to cognitive function, participation in society, caregiver HRQoL and estimated burden. The assessor at the 6-month follow-up was blinded for treatment temperature allocation, but control patients were identified as such.

### Setting

In this sub-study, 20 of the 34 TTM sites located in Sweden, Denmark, the Netherlands, the United Kingdom and Italy participates. The patients attended the follow up at an institution or in their own home/nursing home. The examiner was an occupational therapist, psychologist, a study nurse or a physician. The examiners were trained at specific trial meetings and written manuals were distributed. A centralized support-service and monitoring function was available throughout the study to prevent missing data.

### Ethical approval, informed consent and trial registration

Ethical applications for the TTM-trial and the present cognitive sub-study have been approved in all participating countries. Written informed consent to participate in the sub-study was obtained before the testing, 6 months after the arrest. The TTM-trial and this sub-study are registered at ClinicalTrials.gov (Identifier: NCT01020916 and NCT01946932).

### Study population

**Figure 1:**
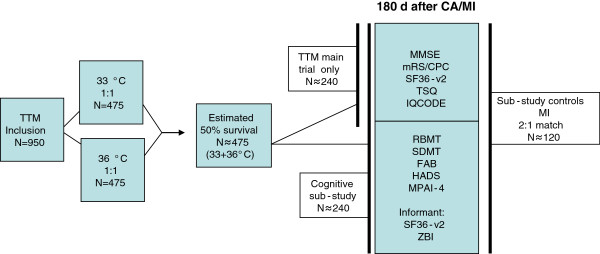
Predicted study inclusion in main trial, sub-study and control group.

The TTM-trial included adult, unconscious patients, with sustained return of spontaneous circulation after cardiac arrest, of a presumed cardiac origin. We estimated that approximately 50% of patients included in the trial would survive until 180 days follow-up [19]. Patients were centrally randomized through a web-based system to target temperature management at 33°C or 36°C target temperature treatment [19]. This sub-study included all TTM survivors at the participating sites that met the inclusion criteria and who lacked exclusion criteria, with a retained 1:1 randomization to the two temperature arms since randomization was stratified for site (Figure 1). For each patient included in the sub-study an informant was asked to participate. The informant is defined as a relative or a close friend with the potential to act as a caregiver if needed.

A control group was recruited at 1:2 ratio for the CA-patients (one control for two CA patients) from a cohort of patients with ST-elevation myocardial infarction and emergency percutaneous coronary intervention. The controls were matched to the cardiac arrest patients for country, age (best match), gender and time-point of hospitalization (± 2 weeks). All recruitment and follow-up of controls was centralized to one centre in each participating country. Also for the controls, an informant was included.

### Exclusion criteria

An exclusion criterion for the TTM main trial is a pre-arrest CPC-score ≥ 3 [19]. An additional exclusion criterion for the sub-study is the inability to speak the local language well enough to complete the tests without an interpreter. The exclusion criteria for the control patients are the same as for the TTM patients, and also that they should not have sustained a cardiac arrest.

### Socio-demographical and medical variables

Socio-demographic and medical variables was obtained at the follow-up or from the TTM database (Table 1). Information about age, gender and the frequency of contact with the patient was collected from the informant.

**Table 1:** Socio-demographical and medical variables

•**Age**	
•**Gender**	
▪**Co morbidities**	
	▪Chronic heart failure (NYHA 3 or worse)
	▪Previous acute myocardial infarction
	▪Ischemic heart disease
	▪Previous cardiac arrhythmia
	▪Arterial hypertension
	▪Previous neurological disease
	▪Diabetes mellitus
	▪Asthma or chronic obstructive pulmonary disease
•**Pre-hospital variables (only CA patients)**	
	▪Location of cardiac arrest
	▪Bystander witnessed arrest
	▪Bystander cardiopulmonary resuscitation (CPR)
	▪First monitored rhythm at arrival of emergency medical service
	▪Use of active compression-decompression device
	▪Time from cardiac arrest to start of basic life support
	▪Time from arrest to start of advanced life support
	▪Time from arrest to return of spontaneous circulation
	▪Cardiac interventions
	▪Percutaneous coronary intervention
	▪Coronary bypass grafting
	▪Valvular surgery
	▪Implantable cardioverter-defibrillator
	▪Pacemaker
•**Hospital length-of-stay**	
•**Pre-arrest Cerebral Performance Category (CPC)**	
•**Level of education (≥12 years/<12 years)**	
•**Medications at the time of the arrest (MI)**	
•**Medications at the time of the follow-up**	
•**Disabilities that could affect the test performance**	
	▪Hearing
	▪Speech
	▪Dyslexia
	▪Vision
	▪Paresis
•**Time from CA/MI to follow-up**	
•**Employment status (pre-arrest/follow-up)**	
•**Place of stay at the time of follow-up**	

### Outcome measures in the main TTM trial

All patients alive at 6 months in the TTM trial were followed up as previously described [19]. Tests and questionnaires of the main trial were presented prior to tests and questionnaires of the sub-study. The entire procedure requires approximately 60–120 minutes (Table 2).

**Table 2:** Tests and questionnaires for the TTM main trial and cognitive sub-study

**Instrument**	**Abbreviation**	**TTM Main trial**	**Sub study**	**Area**	**Test administration**			
					**PS**	**PO**	**IS**	**ER**
Short Form Questionniare-36 version 2	SF-36v2©	■	■	Health related quality of life	■		■	
Two Simple Questions	TSQ	■		Everyday activities/cognition	■			
Informant Questionnaire on Cognitive Decline	IQCODE	■		Cognition			■	
MiniMental Status Examination	MMSE	■		Cognition		■		
Cerebral Performance Category	CPC	■		General neurological outcome				■
modified Rankin Scale	mRS	■		General outcome				■
Rivermead Behavioural Memory Test	RBMT		■	Memory		■		
Symbol Digit Modalities Test	SDMT		■	Attention/Concentration/Mental speed		■		
Frontal Assessment Battery	FAB		■	Executive functions		■		
Hospital Anxiety and Depression Scale	HADS©		■	Anxiety/Depression	■			
Mayo-Portland Adaptability Inventory-4	MPAI-4		■	Impairments/Adjustment/Participation	■			
Zarit Burden Interview	ZBI©		■	Caregiver burden			■	

PS= Patient Subjective PO= Patient Objective IS= Informant Subjective ER= Examiner rating.

*Cerebral Performance Categories scale (CPC)*[20] is considered the gold standard for neurological outcome in resuscitation trials [21]. It is a crude ordinal scale to measure outcome status and performance due to neurological function.

*modified Rankin Scale (mRS)*[22], is a similar scale as the CPC but more directed towards global disability. It focuses on dependence in ADL as well as residual symptoms and is considered to be able to detect even mild symptoms. Examiners in the TTM study were encouraged to use a web based video training [23,24] and the simple algorithm described by Rittenberg [25].

*The MiniMental Status Examination (MMSE)*[26] is the most widespread cognitive screening test available. It covers 8 cognitive domains (orientation, registration, attention, recall, language, reading, writing and drawing). In the TTM study, the item serial 7 was used to test attention since it is more sensitive to detect frontal lobe dysfunction [27].

**Figure 2:**
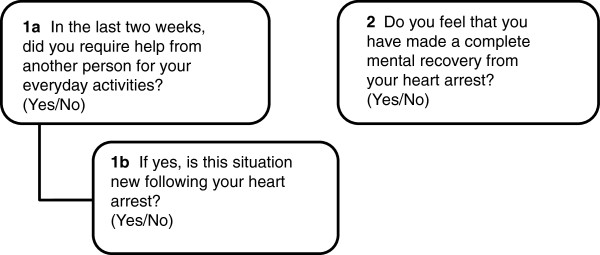
Two simple questions (TSQ).

*Two Simple Questions (TSQ)* was developed for self-estimation of outcome in stroke survivors, but was modified to suit a CA population [28]. In a prior study, the TSQ correlated with the results of cognitive screening and self-assessed HRQoL, but it was insensitive to pre-arrest ADL dependency [28]. We therefore decided to extend the TSQ with question 1b (Figure 2).

*Informant Questionnaire on Cognitive Decline (IQCODE)*[29] is an instrument to detect cognitive decline reported by an informant. It has not previously been used for CA-patients and for this study we obtained permission from the author of the questionnaire to modify the original “compared to 10 years ago” to “compared to before the cardiac arrest”.

*Short Form Questionnaire© - 36 items, version 2 (SF-36v2)*[30] is a Health Related Quality of Life (HRQoL) questionnaire. All surviving patients in the main trial completed the SF-36v2©. Additionally, in the cognitive sub-study the informants completed SF-36v2© to measure their HRQoL.

### Additional outcome measures in the cognitive sub-study

*Rivermead Behavioural Memory Test (RBMT)*[31] focuses on memory skills needed in everyday life. The RBMT is available in several versions but the sub-study used the original version since it was available in the language of all participating countries.

*Symbol Digit Modalities Test (SDMT)*[32] is a test for mental speed, attention and concentration. The SDMT is available in a written and an oral form that could be administered in parallel, this sub-study used mainly the oral form.

*Frontal Assessment Battery (FAB)*[33] is a bedside screening instrument for executive/frontal lobe dysfunction shown to correlate with more traditional tests measuring single executive functions [34].

*Hospital Anxiety and Depression Rating Scale© (HADS)*[35] is a questionnaire shown to be well suited to identify anxiety and depression [36] and is commonly used for this purpose in CA studies [37].

*Mayo-Portland Adaptability Inventory-4 (MPAI-4)*[38] is a questionnaire based on the WHO’s international classification of functioning, disability and health. It measures different aspects of physical, cognitive, emotional, behavioural and social problems following brain injury, as well as obstacles to community integration and social participation. It could be completed by the patient, an informant or staff, all with similar levels of reliability [39]. In this study, the patients rated their outcome (item 1–29), with the exception of the severely impaired, where the informants completed the test.

*Zarit Burden Interview©*[40] is a 22 point scale that is completed by the informant. It was developed for dementia care but is suitable for use by all caregivers of cognitively impaired older adults [41].

### Statistical analysis and sample size calculation

In the primary analyses of cognitive function between the two treatment groups and between the two treatment groups and controls, three comparisons will be made (RBMT, FAB, and SDMT). To correct for multiple comparisons, the Bonferroni-Holm method with a critical p-value of 0.017 will be used. If the data are normally distributed or possible to transform to normal distribution, the t-test and ANOVA will be used. If a normal distribution cannot be obtained, we will apply non-parametric test methods (Wilcoxon Mann Whitney/Kruskal Wallis). Descriptive statistics will be used for the baseline characteristics of the sub-study participants (treatment groups and controls), the whole TTM population and patients not-included the sub-study.

Initial randomization will be maintained by inclusion of non-responders (CPC score including dead) in the first comparison between the two temperature arms. We will use multiple imputations for missing values to prevent bias due to incomplete or absent test results from the most severely impaired patients. In the comparison between the two treatment groups and controls, only survivors will be included. Confounders will be adjusted for by multiple regression.

In a previous case–control study [6] the RBMT score was 20% lower in the CA population. To correctly identify a 10% difference (2.4/24points on RBMT, SD 5 [6]) between the two temperature arms, with the type I error probability set to 0.017, we estimated a need for 100 patients in each treatment group and a power of 83,5%. There are no similar studies with FAB and SDMT.

Cognitive impairment measured by the three tests: RBMT, FAB and SDMT will be correlated to participation in society (MPAI-4/return to work), HRQoL for patients and caregivers (SF-36v2©), and caregivers feeling of burden (ZBI©).

### Trial status and time-line

Enrolment in the TTM-trial started November 2010 and inclusion stopped at target 950 patients January 2013. The first follow-up of this sub-study was performed in June 2011 and the last patient is estimated to be included September/October 2013. Analyses of the sub-study outcome measures will commence after publication of the main trial results which is expected to be in November 2013.

## Discussion

Global brain injury and cognitive dysfunction are well described consequences of cardiac arrest but the prevalence of impairment and the consequences for patients and their relatives have never before been described in a large international cohort. Utilising a relevant control-group with similar risk factors for cognitive impairment our study will also add information on the prevalence of cognitive problems among patients with myocardial infarction only. The actual impact of the CA will be expressed as the difference between CA patients and controls and is likely to be considerably smaller than in previous trials without a control-group.

The primary aim of the TTM-trial is to assess whether survival is affected by systemic cooling to 33°C compared to a controlled temperature of 36°C. In addition, neurological outcome is compared between the two treatment groups by the established but crude outcome scales CPC and mRS. The CPC-scale is best suited to identify moderate to severe impairment and has less ability to differentiate in the higher end of none to mild impairment [3,15]. This sub-study will add systematic cognitive testing in survivors from the two temperature arms with the original randomization maintained. Consequently more subtle neuroprotective effects of systemic cooling may be revealed.

This sub-study is an extension of the follow-up platform that we use in the TTM-main trial. In order to enable follow-up of all patients in this large international intensive care trial, we choose tests that were possible to perform in a short period of time, with limited training and support, and by examiners from different healthcare backgrounds.

The simple test battery of the TTM main trial is based on a model that has been in use in one of our institutions in recent years. Our experience is that this test battery is easily integrated as a screening tool during normal ICU follow-up and sensitive enough to identify the majority of patients who have significant cognitive problems. Although the MMSE test has known ceiling effects and may be insensitive to mild to moderate cognitive difficulties [3], our hypothesis is that the combination of objective (MMSE), subjective (TSQ) and proxy ratings (IQCODE) will increase the ability to identify patients with cognitive impairment in the main TTM trial. An advantage with the IQCODE is that it can be used for patients who are unable to participate in cognitive testing [42].

Instruments for the assessment of cognitive function in the sub-study were chosen to assess the cognitive domains mostly affected by CA (memory, attention, executive functions) [3]. They have previously been used in similar studies [6,43-46] and are all easy to administer with some training. In addition, we added tools for more subjective areas of participation in society and caregiver’s health and judgements of burden.

In a small study by Middlekamp and co-workers [9], it has been proposed that participation in society may be affected by cognitive disability due to CA. However, cognitive impairment may have different effects on participation in society according to which activities, roles and environment the patient needs to return to [47]. The large sample size of this sub-study will substantially increase the available information regarding this association.

Cognitive dysfunction may be perceived as a greater problem by caregivers than amongst affected patients e.g. due to lack of insight. This may lead to feelings of increased burden and poor health amongst caregivers [5]. Relatives to CA patients were found to have increased levels of anxiety [4] and decreased HRQoL [5]. Moreover, family members of critically ill patients in general may have psychological distress due to posttraumatic stress [48]. Whether caregiver distress is related to the degree of cognitive impairment in patients has not previously been investigated.

We choose to perform the follow-up at 6 months. At this time-point most of the spontaneous recovery has occurred, and any dysfunction that remains will be long term [49,50]. Since it is impossible to estimate the precise pre-arrest cognitive function of our patients, we use a matched control group to evaluate the influences of cardiac arrest according to previous recommendations [51,52]. Patients with myocardial infarction were found to lack significant long-term memory impairments [6,53,54], but to have a similar pattern of HRQoL as patients having suffered cardiac arrest [55]. In addition, they share the same cardiovascular risk factors and risk of mood disturbances due to acute hospitalization, which makes them a relevant population to study as controls.

Mild to moderate cognitive impairment in a CA-survivor may easily be overlooked by treating physicians [50] as these patients are more often regarded as having cardiological rather than neurological disease [4]. The simple cognitive screening model used in the TTM trial is easy to apply in any clinical setting and with minimal resource utilization. The results from this sub-study could be used to evaluate whether the suggested screening model is able to identify patients at risk. There is a clear need for a structured approach to identify CA-patients with cognitive problems [50,56,57] and to increase the knowledge about how these will affect patients and their families, so clinicians can target appropriate support. If the screening model proves to be accurate/specific enough it could have a large clinical impact as a consequence of its simplicity. This study will generate important information concerning neurological outcome and quality of life for CA survivors and their families, as well as the impact of temperature management.

## Competing interests

The authors declare no financial or non-financial competing interests.

## Authors’ contributions

GL, TC and NN designed the sub-study. GL is the coordinator of the trial and TC the principal investigator. NN, JK, JH, MW and TP are national investigators. FN is the trial statistician. GL and TC drafted the manuscript that was read, revised and finally approved by all co-authors.

## Pre-publication history

The pre-publication history for this paper can be accessed here:

http://www.biomedcentral.com/1471-2261/13/85/prepub
